# A Rare Case of Calculous Gallbladder Hydrops Presenting With Atypical Abdominal and Urinary Symptoms

**DOI:** 10.7759/cureus.40016

**Published:** 2023-06-05

**Authors:** Zhuang Hui Mark Le, Lachlan Dowling, Sarangi M Ranasinghe

**Affiliations:** 1 Department of General Surgery, Redland Hospital, Brisbane, AUS; 2 Department of General Surgery, Logan Hospital, Brisbane, AUS

**Keywords:** surgery general, cholecystectomy laparoscopic, acute calculus cholecystitis, gallbladder mucocele, gallbladder hydrops

## Abstract

Gallbladder hydrops, otherwise known as gallbladder mucocele, is an uncommon gallbladder condition characterised by gallbladder distention and accumulation of clear mucous-like inspissated bile. Patients with gallbladder hydrops are usually asymptomatic and incidentally diagnosed on cross-sectional imaging or diagnostic laparoscopy. This case report presents a rare case of calculous gallbladder hydrops measuring 217mm in maximal length in a 56-year-old female presenting with atypical abdominal and urinary symptoms. The radiological and intraoperative images will also be presented to highlight the extensiveness of the disease and therefore the importance of considering gallbladder hydrops as a differential in these patients.

## Introduction

Gallbladder hydrops is an unusual gallbladder pathology characterised by gallbladder distention and accumulation of clear mucous-like inspissated bile [1]. It is caused by chronic obstruction of the cystic duct or distal biliary tree often secondary to obstructed gallstones and concurrent cholecystitis [2]. It is usually diagnosed incidentally radiologically or intraoperatively [3]. This case report presents and discusses the atypical clinical presentation, impressive radiological and intraoperative images and surgical management of a 56-year-old female with a rare calculous gallbladder hydrops extending 217mm in maximal length.

## Case presentation

A 56-year-old female presented to the emergency department with a four-day history of gradual-onset constant lower abdominal pressure, aggravated on movement and when laying on her right side. This was associated with occasional nausea, dyspepsia and some urinary urgency, but no vomits, anorexia, changes in bowel habits, or constitutional symptoms. She had no significant medical or surgical history and denied smoking, alcohol or recreational substances. A large mass was palpable, extending from the right upper quadrant to the right lower quadrant. No other clinically significant findings were elicited on examination.

**Figure 1:**
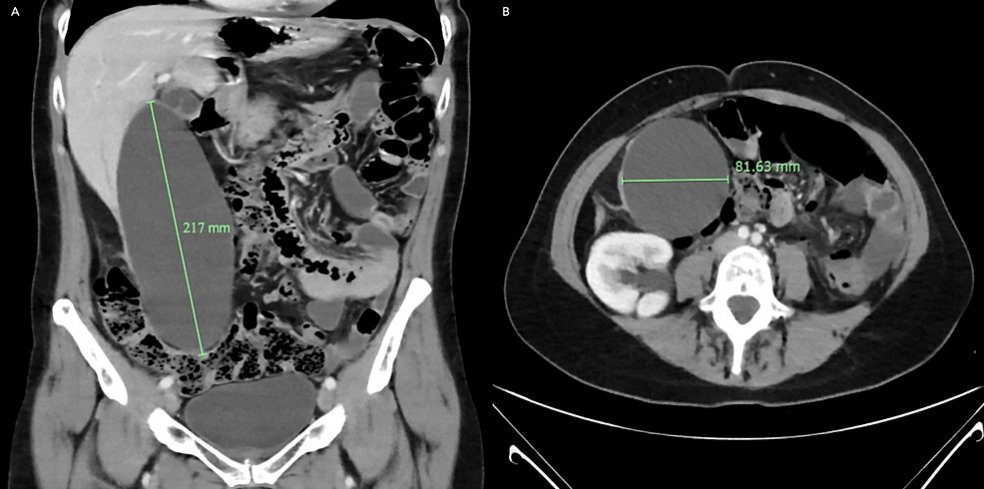
CT images demonstrating a significantly distended thin-walled gallbladder with a (A) maximal cephalocaudal extension of ~217mm and (B) maximal transverse diameter of ~81mm.

Haematological investigations demonstrated normal inflammatory markers and unremarkable liver function tests (LFT) (Table 1). An ultrasound (US) scan of the abdomen showed a markedly distended gallbladder extending to the right iliac fossa with a length of ~200mm and up to 10 mobile non-obstructive gallstones, but no gallbladder wall thickening, no common bile duct dilatation (~5mm) and no features of pancreatic pathology. Only an incidental 5mm well-defined cyst-like hypoechoic focus in segment VIII of the liver was noted. A computed tomography (CT) scan of the abdomen and pelvis with intravenous contrast further showed a significantly distended gallbladder with a maximal cephalocaudal extension of ~217mm and a maximal transverse diameter of ~81mm (Figures 1A, 1B). The CT scan demonstrated no gallstones, normal gallbladder wall thickness (~3mm) and no extrinsic obstructive lesions. However, the common bile duct was reported with a maximal dilatation of ~10mm without intraductal stone or sludging.

**Table 1:** The patient's most pertinent haematological investigations.

Pertinent Haematological Investigations	Reference Range	Patient Results
White Cell Count (WCC)	4.0 - 11.0 10^9^/L	9.3 10^9^/L
C-Reactive Protein (CRP)	< 5mg/L	< 0.5 mg/L
Conjugated Bilirubin	< 20 µmol/L	11 µmol/L
Unconjugated Bilirubin	< 4 µmol/L	4 µmol/L
Alkaline Phosphatase (ALP)	30 - 110 U/L	60 U/L
Gamma-Glutamyl Transferase (GGT)	< 38 U/L	16 U/L
Alanine Transaminase (ALT)	< 34 U/L	25 U/L
Aspartate Transaminase (AST)	< 31 U/L	39 U/L
Lipase	< 60 U/L	43 U/L

**Figure 2:**
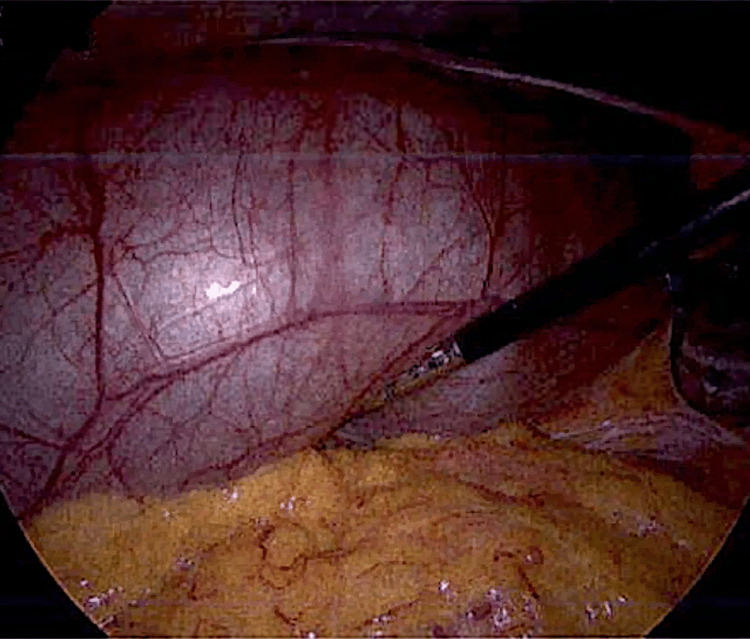
Intraoperative images demonstrating an injected, distended and thick-walled gallbladder extending from the right subhepatic fossa to the greater pelvic cavity.

Subsequent laparoscopic cholecystectomy with intraoperative cholangiogram was performed (Figure 2). The gallbladder was first decompressed laparoscopically, with clear mucous-like fluid suctioned, before undertaking a routine and uncomplicated cholecystectomy. The intraoperative cholangiogram demonstrated a mildly dilated common bile duct but otherwise normal biliary tree contrast flow with no strictures or obstructive stones. The gallbladder histopathology showed cholelithiasis and mild chronic inflammation with thickened muscularis propria and Rokitanksy-Aschoff sinuses and attenuated mucosa with underlying fibrosis. There was no evidence of microscopic dysplasia or malignancy. The patient’s symptoms resolved post-operatively and she was discharged home two days post-cholecystectomy.

## Discussion

Gallbladder hydrops is an uncommon condition characterised by gallbladder distention due to inappropriate accumulation of mucous or inspissated bile [1]. It is caused by prolonged obstruction of the cystic duct or distal biliary tree, often secondary to cholelithiasis and cholecystitis (as was this patient's case) [2]. Rarer aetiologies include tumours at the gallbladder neck or common bile duct (e.g., gallbladder polyps, cholangiocarcinoma or pancreatic carcinoma), inflammatory conditions (e.g. Kawasaki disease) and extrinsic compression of the biliary tree (e.g., adhesions and congenital strictures) [2]. Bile stasis from prolonged obstruction results in bacterial colonisation and an increase in intraluminal pressure, leading to gallbladder wall distention, ischaemia and inflammation [3].

As such, gallbladder hydrops oftentimes present as acute cholecystitis [3,4]. However, patients can also present with chronic right upper quadrant abdominal pain, bloating, early satiety, nausea/vomiting and a palpable gallbladder [3,4]. These features can be present for many years before diagnosis. This case demonstrated an atypical presentation of gallbladder hydrops with the patient experiencing predominantly mass-effect-related symptoms of lower abdominal pain, dyspepsia and urinary urgency. It was hypothesised that these symptoms were caused by compression of the extremely distended gallbladder on the stomach, small and large bowel and secondarily the urinary bladder (that is, compression of small and large bowels onto the urinary bladder). Upon review of the literature, no case reports to date have presented gallbladder hydrops causing mass effects on the urinary bladder.

Gallbladder hydrops is diagnosed with a combination of clinical features, radiological investigations and intraoperative findings. Blood is usually unremarkable unless a concurrent gallbladder pathology is contributing to symptoms (e.g., an elevated white cell count and C-reactive protein in acute cholecystitis, deranged cholestatic liver function tests in choledocholithiasis, elevated lipase in pancreatitis or pancreatic malignancies) [5]. A US or CT would demonstrate a distended and oedematous gallbladder with mobile or impacted non-mobile gallstones in the cystic duct or Hartman pouch [5]. Gallbladder hydrops is frequently undiagnosed before surgery and is, instead, incidentally discovered during laparoscopic cholecystectomy [3]. It is during decompression of the gallbladder intraoperatively, when clear mucous-like fluid instead of green or brown bile is suctioned, that a diagnosis of gallbladder hydrops is often made [3].

The recommended management for gallbladder hydrops is laparoscopic decompression of the gallbladder followed by routine cholecystectomy [6]. This is because the disease is often recurrent and laparoscopic intervention has low morbidity and mortality with quick recovery [6]. Alternatively, if patients are poor surgical candidates, temporary image-guided percutaneous drainage of the gallbladder can be performed [7].

## Conclusions

Gallbladder hydrops is an uncommon gallbladder condition caused by chronic cystic duct obstruction and is often diagnosed incidentally radiologically or intraoperatively. It is an important differential of abdominal pain associated with non-specific urinary and gastrointestinal symptoms due to the commonness of its primary aetiology - cholelithiasis and subsequent cholecystitis. This case demonstrates the rare case of an enormous calculous gallbladder hydrops measuring ~217mm in maximal length and an atypical presentation with acute non-specific abdominal symptoms and without any usually associated features of cholecystitis. Lastly, this case exhibits the importance of radiological investigations and intraoperative findings for the diagnosis and treatment of gallbladder hydrops.
